# Sensitive Dentine

**Published:** 1893-06

**Authors:** W. G. Beers

**Affiliations:** Montreal.


AMERICAN JOURNAL

—OF—

DENTAL SCIENCE.

VOL. XXVII. BALTIMORE, JUNE, 1893. NO. 2.

ARTICLE I.

### SENSITIVE DENTINE.

BY W. G. BEERS, L. D. S., MONTREAL.

Vermont State Dental Society.

A society of savants was once organized in Germany, with the explicit rule that every paper read before it was to be absolutely and infallibly original. It met twice, then died for lack of material. It occurs to me that if we were gathered here upon the same condition, my paper, at least, would be unconstitutional, as it is almost impossible, without being impracticable, to contribute anything new on the subject of Sensitive Dentine. And yet are we not apt to forget that some points may be, or must be new to somebody, and that even old facts are never too often repeated, if they have not been sufficiently learned. Were dentine no more sensitive than enamel, what a boon it would be to our patients, what an immense blessing to ourselves! what pathological consequences would be avoided! One may, therefore, venture to prowl about, and into old and well-beaten tracks.

What do we mean when we speak of sensitive dentine? If you examine dentine, you will discover that, strictly speaking, we do not mean what we say. Macroscopically we observe that it is a structureless matrix impregnated with lime salts, and that if the enamel and cementum were entirely removed, the tooth would still retain its form and character. Any pretence that the matrix *per se* is living protoplasm, or that it can in any way display vital phenomena, is a hypothesis incapable of proof. When now we examine the tissue microscopically, we see that from the pulp cavity to the periphery, the entire dentine is perforated with numberless small tubes or canals, having distinct walls; each tube starting by an open circular mouth upon the surface of the pulp cavity, radiating in an undulating course, giving off many branches which freely anastomose or communicate with each other, something like the arteries and veins of the body; but which do not reach the periphery of the dentine, as each tube becomes smaller and breaks up into branches at a little distance beneath the surface of the dentine. Sometimes, as an anomaly, these tubes pass into the enamel and cementum. Each tube has a definite wall or lining that may be demonstrated, even in fossil teeth. The tubes are not mere bony canals or ducts in the matrix, like pipes put through chalk, but each tube is lined with a definite and delicate, and yet indestructible structure, the "dentinal sheath of Neumann." You may boil dentine in caustic alkali; you may reduce it by concentrated hydrochloric acid; you may submit it to putrefaction, and though you destroy the cartilage, and leave it a slimy and shapeless mass, the sheaths of Neumann remain intact. But neither of these structures explain the so-called sensitiveness of dentine. The tubes were once supposed to be solid fibres: afterwards it was thought that they were the conveyers of a nutrient fluid; but Tomes proved that they are occupied by little soft fibrillæ, which, like nerve-filaments, conduct sensory impressions to the pulp. The fibrils are processes, or prolongations of the odontoblasts, which are situated upon the periphery of the pulp, lining the pulp-chamber. No true nerves, or nerve fibrils have ever been demonstrated in dentine; but fine nerve filaments are found close to the odontoblasts, and, as Black shows, they communicate to the sensorium the sensation made on the protoplasm of the odontoblasts through the injury to the fibrils. It is not even necessary to assume that fibrils are nerves, before recognizing that they can communicate sensation. Many animals, which have no demonstrable nervous system, are endowed with sensation. As Black shows, protoplasm in itself may be sensitive, as is seen in the amœba, the leucocyte, etc., which respond to stimulants and exhibit sensitiveness to thermal changes.

However, there is no doubt but that the sensitiveness is due to the presence of the tube contents, whether nerve fibres enter or not. We are still ignorant of a great deal as to the peripheral distribution of the nervous system, and it would be rash to say that we know all about the nerve structure of the tooth. Only recently a new addition was made to our knowledge of muscular tissue, and it is quite certain, that if any of us live ten years longer, we will know a great deal we do not know now as to the structure, mode of action and functions of the more complex nervous system.

Sensitiveness of dentine is purely physiological, but we cannot assert that it is never pathological. It seems reasonable to believe that a pathological condition of the fibrils at once follows fracture of dentine, or a severe blow, which the pulp resents but cannot resist. Yet it is a demonstrable fact that the teeth differ as to this susceptibility the same as nerve, muscular and other soft tissues: that not only is there a great variation in different mouths, but that the more inexplicable fact presents itself, that in the same mouth at the same time, in teeth apparently under the same conditions as to the extent of caries, there are frequently remarkable differences in degree during excavation. This, to me, is quite different, and more obscure, than the fact that the greatest sensibility is at the point of ultimate distribution of the dentinal tubes and their contents—that is, immediately below the enamel. This latter fact would alone prove that the fibrils are organs of sensation, and subject to the same laws as nerves of sensation, the highest sensibility of which is confined to their terminal branches. How do we explain sensitiveness in one cavity in the same mouth more than in another cavity in the tooth beside it? If it was on the opposite side, we might perhaps say that it was due to the concentrated action of a chemical agent, which has a stronger affinity for the fibrils than for the dentine; but that would not explain the difficulty.

The dentine of rapidly decaying teeth is more sensitive in slow caries. A fracture which exposes the dentine is hypersensitive at the line of fracture. Frequently we find in worn down crowns a point intensely sensitive, so extremely minute that we cannot diagnose it with a probe. Newly opened, and especially obscure cavities, are more sensitive than cavities of the same size and age which have been exposed to mastication. For this reason, approximal cavities are more sensitive, as a rule, than those on the crowns; and those bordering on the cementum more sensitive than either, especially when, through the medium of the "granular" layer of the dentine, there happens to be a fusion of the two. Frequently the sensitiveness is confined to the layer of decomposed dentine which we scoop out at a cut with a spoon excavator. Why is this decomposed layer *per se* so hyper-sensitive? You can put that in the question box for some one to answer.

During menstruation and pregnancy, and in many constitutional conditions, especially in rheumatism, gout, etc., when there is an acid reaction, there is an exalted sensibility of the fibrils. It is a fact that the fibrils, which were intensely sensitive before exposure of the pulp, over their layer of dentine, decomposed or not, are reduced by actual exposure.

Whatever controversy may exist as to the histology and physiology of this subject, we know that the preservation of the normal integrity of the fibrillæ is important, excepting, perhaps, in old teeth. In children's teeth, especially in the deciduous set, the fibrillæ are generally hyper-sensitive, and it is unwise to place a metal filling over the dentine, without the interposition of a non-conductor, or, at least, carbolizing the albumen of the cavity by previously inserting carbolic acid for a few minutes, or by following the idea presented by our friend, Dr. Stebbins, in the use of nitrate of silver.

Hyper-sensitiveness may be so intense that constitutional treatment may be advisable. It may be associated with extreme sensitiveness of bodily and mental condition, a high-strung intensity of the nervous system, which is so common in this country that we scarcely suspect it of being pathological. You know there are patients who will scream, if they do not faint, over a simple excavation, while others are fearless, and, in fact, would not suffer in proportion in an amputation. We find the same fact with the lower animals. If you prick one horse with a pin, he will bolt; you may kick another, if you are a brute yourself, and he will think more than twice before he stirs.

I suppose we are all more practically concerned in the treatment of sensitive dentine than in questions of its physiology. The dental pharmacopœia is full of suggestions from the earliest times. That our materia medica of the present day is by no means an entire novelty, may be seen by anyone who reads Chap. ix., Book vi. of A. Cornelius Celsus, who flourished in the time of Tiberius III., Emperor of Rome. When we discuss the therapeutics and materia medica of dentistry, let us be humbled by the reflection that over seven hundred years before the United States was a nation, the ancients had investigated and discovered remedies many of which are still in use.

Among the applications tested for relief, may be mentioned all the narcotics, anæsthetics, sedatives, and escharotics. Having said that, it might not seem necessary to particularize, but some have had more success than others; some have proved a delusion and a snare. None have, perhaps, done more mischief than arsenic—a comfort in disguise. Various combinations of creosote, carbolic acid, chromic acid, tannic acid, chloride zinc, nitrate of silver, cobalt, chloral, cocaine. Chromic acid, and chloride of zinc give severe pain on account of their greater affinity for water, and are destructive if used near the pulp. Nitrate of silver in crystal, or as a saturated solution, is useful at the periphery of the dentine, but if near the pulp it may also cause its death. Herbst's obtundent is simply a saturated solution of cocaine hydrochlorate in chemically pure sulphuric acid, to which is added sulphuric ether to the point of saturation, allowing the excess of ether to escape by evaporation. Even that must be used with caution. If there is no excess of acid, use alkali mouth washes, holding a drachm of carbonate of soda in the mouth for a few minutes frequently during the day. Rapid wedging, by forcing the apex of the root against the foramen portion of the nerve, partly choking circulation and obstructing connection with the sensorium, has a remarkable benumbing influence on the fibrillæ, which are temporarily strangulated. Rapid cutting with sharp excavators or burrs, exhaust the fibrils by frequent irritation. Half the pain given by many operators is due to poorly tempered and badly sharpened instruments. Dehydration, or drying the cavity by absolute alcohol, keeping on the rubber dam, the use of hot air, with various preparations of tannin, etc., using temporary fillings to give physiological rest to the fibrillæ, filling the cavity lightly with cotton, and applying, with an assistant, rhigolene spray, or in extreme cases of necessity, administering nitrous oxide, and operating rapidly during general anæsthesia. That is all, I think, I know. Perhaps we may yet discover something in the application of electricity.

In conclusion, I must say that I have an instinctive dislike to the use of the term "sensitive dentine." It is one of those many inaccuracies of expression and definition for which the nomenclature of dentistry is distinguished. We have, in many instances, as many names for one meaning as Mahomet had floors in his heaven, and the student who follows the dental literature of modern times will discover, that every writer is a law unto himself, to make or murder etymology, and to render Greek and Latin derivatives into that sort of English which Dr. Johnson defined, when speaking of poetry, as "ingenious nonsense." As the different points of the compass have a local wit and wisdom of their own, so they curiously seem to have an independent scientific terminology. There is so much poor coining of words in dental science, and so little harmony in their use, both by teachers and text-books, that the fashion of pitching new terms into our vocabulary is becoming a contagious nuisance. Somebody founded in Italy the *Academia della crusca*, a society for restoring purity to the native language. Some day—let us hope at the World's Dental Congress—a special section will be entrusted with the duty of definitely settling our definitions.

Dentine *per se* is no more sensitive than enamel. We do not mean what we say when we speak of sensitive dentine; then, why do we not say what we mean? There is no such condition or possibility as sensitive lime salts. It may be thought that this is splitting hairs in argument to disapprove of terms which custom so long has sanctioned; but custom has no mortgage on fact. It may be said we call the epidermis "sensitive," but it is only the nerve filaments that makes it sensitive. Paralysis destroys the sensation of touch; a palsied hand will respond to stimuli by reflex action without sensation. When the nerve filaments are palsied, the skin *per se* is as dead to sensitiveness as if it were parchment. Every one knows that when the pulp dies, the dentine loses its so-called sensitiveness, and that with living pulps it is often no more sensitive in excavation than the enamel. Dentine would not be sensitive if the branch of the nerve leading to the tooth was severed. Yet, in those several conditions there would be no alteration in the chemical or microscopical character of dentine. We know very well that the sensitiveness is due to the contents of the tubuli, which transmit sensation to the pulp. Dentine is nothing but the passive matrix, in which lie the sources of sensation. Therefore, logically as well as scientifically, the term sensitive dentine is a misnomer, and we should say "sensitive fibrillæ." Perhaps we should not.—*Dominion Dental Journal.*

